# Nicotinamide Riboside Kinase Structures Reveal New Pathways to NAD^+^

**DOI:** 10.1371/journal.pbio.0050263

**Published:** 2007-10-02

**Authors:** Wolfram Tempel, Wael M Rabeh, Katrina L Bogan, Peter Belenky, Marzena Wojcik, Heather F Seidle, Lyudmila Nedyalkova, Tianle Yang, Anthony A Sauve, Hee-Won Park, Charles Brenner

**Affiliations:** 1 Structural Genomics Consortium and Department of Pharmacology, University of Toronto, Toronto, Canada; 2 Departments of Genetics and Biochemistry and Norris Cotton Cancer Center, Dartmouth Medical School, Lebanon, New Hampshire, United States of America; 3 Department of Pharmacology, Weill Cornell Medical College, New York, New York, United States of America; Stanford University, United States of America

## Abstract

The eukaryotic nicotinamide riboside kinase (Nrk) pathway, which is induced in response to nerve damage and promotes replicative life span in yeast, converts nicotinamide riboside to nicotinamide adenine dinucleotide (NAD^+^) by phosphorylation and adenylylation. Crystal structures of human Nrk1 bound to nucleoside and nucleotide substrates and products revealed an enzyme structurally similar to Rossmann fold metabolite kinases and allowed the identification of active site residues, which were shown to be essential for human Nrk1 and Nrk2 activity in vivo. Although the structures account for the 500-fold discrimination between nicotinamide riboside and pyrimidine nucleosides, no enzyme feature was identified to recognize the distinctive carboxamide group of nicotinamide riboside. Indeed, nicotinic acid riboside is a specific substrate of human Nrk enzymes and is utilized in yeast in a novel biosynthetic pathway that depends on Nrk and NAD^+^ synthetase. Additionally, nicotinic acid riboside is utilized in vivo by Urh1, Pnp1, and Preiss-Handler salvage. Thus, crystal structures of Nrk1 led to the identification of new pathways to NAD^+^.

## Author Summary

Biosynthesis of nicotinamide adenine dinucleotide (NAD^+^) is fundamental to cells, because NAD^+^ is an essential co-factor for metabolic and gene regulatory pathways that control life and death. Two vitamin precursors of NAD^+^ were discovered in 1938. We recently discovered nicotinamide riboside (NR) as a third vitamin precursor of NAD^+^ in eukaryotes, which extends yeast life span without caloric restriction and protects damaged dorsal root ganglion neurons from degeneration. Biosynthesis of NAD^+^ from NR requires enzyme activities in either of two pathways. In one pathway, specific NR kinases, including human Nrk1 and Nrk2, phosphorylate NR to nicotinamide mononucleotide. A second and Nrk-independent pathway is initiated by yeast nucleoside-splitting enzymes, Urh1 and Pnp1. We solved five crystal structures of human Nrk1 and, on the basis of co-crystal structures with substrates, suggested that the enzyme might be able to phosphorylate a novel compound, nicotinic acid riboside (NaR). We then demonstrated that human Nrk enzymes have dual specificity as NR/NaR kinases in vitro, and we established the ability of NaR to be used as a vitamin precursor of NAD^+^ via pathways initiated by Nrk1, Urh1, and Pnp1 in living yeast cells. Thus, starting from the structure of human Nrk1, we discovered a synthetic vitamin precursor of NAD^+^ and suggest the possibility that NaR is a normal NAD^+^ metabolite.

## Abstract 3

Eukaryotic nicotinamide riboside kinase (Nrk) converts nicotinamide riboside to NAD^+^ by phosphorylation and adenylylation. The structures of this enzyme bound to several substrates lead to identification of new pathways to NAD^+^

## Introduction

NAD^+^ functions both as a co-enzyme for hydride transfer reactions and as a substrate for NAD^+^-consuming enzymes including Sirtuins and poly(ADPribose) polymerases [1]. Most fungal and animal cells have redundant pathways for NAD^+^ biosynthesis that consist of a de novo pathway from tryptophan [2] and salvage pathways that utilize the vitamin precursors of NAD^+^, namely nicotinic acid (Na), nicotinamide (Nam) [3], and nicotinamide riboside (NR) [4]. Because NAD^+^ biosynthesis is required for the function of Sirtuins [5–9] and given the evidence that Sirtuins play roles in life span extension [10–12], increased mitochondrial function [13], and energy expenditure [14], there has been a resurgence of interest in NAD^+^-boosting drug therapies and nutritional interventions [1].

NR, a natural product present in milk [4], increases NAD^+^ biosynthesis, increases Sir2-dependent gene silencing, and extends yeast life span via two NR salvage pathways [15]. The first NR salvage pathway depends on NR phosphorylation by a specific kinase, encoded by the products of the yeast and human *NRK1* genes or the human *NRK2* gene [4]. The second NR salvage pathway is Nrk-independent and is initiated by the activity of yeast Urh1, Pnp1 , and, to a slight degree, Meu1, which split NR into a ribosyl product and Nam for resynthesis of NAD^+^ via Nam salvage [15]. Although the second pathway of NR salvage has yet to be investigated in mammalian systems, Pnp1 and Meu1 are the yeast homologs of human purine nucleoside phosphorylase and methylthioadenosine phosphorylase, suggesting that human NR salvage may depend on Nrk1, Nrk2, and Pnp [15].

Na, Nam, and NR have been investigated in an ex vivo model of murine dorsal root ganglion neurodegeneration [16]. Prompted by genetic evidence that increased neuronal NAD^+^ biosynthesis protects against Wallerian degeneration [17,18], NR was shown to be the only NAD^+^ precursor vitamin that protects against axonopathy without engineered overexpression of a biosynthetic gene, apparently because the *NRK2* gene is transcriptionally induced by nerve damage [16].

NR kinases are ∼200–amino acid polypeptides related to human uridine/cytidine kinase 2 [19] and Escherichia coli pantothenate kinase [20]. To establish that yeast Nrk1 and no other enzyme phosphorylates NR in vivo, Saccharomyces cerevisiae mutants without the *QNS1* gene, encoding glutamine-dependent NAD^+^ synthetase [21] were shown to be entirely dependent on NR and Nrk1 for viability [4]. The human homologs Nrk1 and Nrk2 were validated in the same assay [4]. The presence of a human Nrk pathway suggests the means by which the anticancer prodrug tiazofurin [22] may be converted to the toxic NAD^+^ antagonist tiazofurin adenine dinucleotide (TAD).

Although yeast and human Nrk1 and human Nrk2 were purified and characterized with respect to NR, cytidine, uridine, and tiazofurin phosphorylation in specific activity terms [4], the kinetics of nucleoside and nucleoside triphosphate specificity have not been carefully quantified. Here we report the structure–activity relationships for human NR kinases with nucleoside and nucleoside triphosphate substrates. Nrk1 and Nrk2 both strongly discriminate against cytidine phosphorylation by 500-fold in *k*_cat_ */K*_M_. However, Nrk1 effectively phosphorylates NR with ATP or GTP and discriminates against uridine, whereas Nrk2 discriminates against GTP as a phosphodonor but does not strongly discriminate against phosphorylation of uridine. To dissect the structural basis for specificity, we crystallized selenomethionyl human Nrk1 bound to Mg^2+^·ADP and solved the 1.95 Å structure of the Nrk1 monomer by single-wavelength anomalous scattering. Using a series of crystal structures of human Nrk1 bound to NR, NR·Mg^2+^·adenosine-5′-[(β,γ)-imido]triphosphate (AppNHp), and nicotinamide mononucleotide (NMN), we resolved snapshots of the catalytic cycle and identified two conserved carboxylate groups that we establish as essential for biological activity. From a structure of Nrk1 bound to tiazofurin, we gained further understanding of nucleoside specificity. However, at the site where we expected to find specific enzyme features that would recognize the distinctive carboxamide portion of NR and tiazofurin substrates, we found only steric complementarity and solvent exposure. Accordingly, we synthesized nicotinic acid riboside (NaR) and found this molecule to be as specific a biochemical substrate as is NR. Finally, we showed that NaR is a synthetic vitamin precursor of NAD^+^ that supports the growth of yeast cells through each of the salvage pathways—Nrk and Urh1/Pnp1—also used by NR. Thus, NR kinases are actually dual-specificity salvage enzymes that may play a role in another unanticipated biosynthetic pathway to NAD^+^.

## Results

### Substrate Specificity of Recombinant Nrk1 and Nrk2

To assess Nrk specificity in vitro, recombinant human Nrk1 and Nrk2 were expressed in E. coli and purified by immobilized metal chelate affinity chromatography. As shown in Table 1, the enzymes discriminate between substrates almost entirely in the *K*_M_ term. Nrk1 has a *k*_cat_ of approximately 0.5 s^−1^ irrespective of substrate, and Nrk2 possesses a *k*_cat_ of approximately 1 s^−1^ irrespective of substrate. Nrk1 strongly favors NR as a substrate, displaying a 340-fold preference for NR over cytidine in the *K*_M_ term and a ∼500-fold preference over either cytidine or uridine in the *k*_cat_/*K*_M_ term. Tiazofurin, the prodrug form of the toxic NAD^+^ analog TAD, is a relatively good Nrk1 substrate with a *k*_cat_/*K*_M_ of 1300 s^−1^M^−1^, which represents 19% of the second-order rate for NR conversion to NMN (6800 s^−1^M^−1^). Moreover, Nrk1 shows little preference for ATP (6800 s^−1^M^−1^) over GTP (5000 s^−1^M^−1^) as phosphodonor in formation of NMN.

**Table 1:**
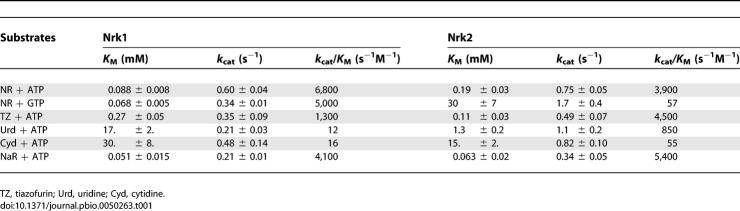
Substrate Specificity of Human Nicotinamide Riboside Kinases

Whereas these data would classify Nrk1 as an NR and tiazofurin:ATP or GTP kinase, the data establish Nrk2 as an ATP-specific NR, tiazofurin, and uridine kinase. As shown in Table 1, with GTP as the phosphodonor, Nrk2 has only 1.5% of the NR phosphorylating activity with respect to ATP. Tiazofurin (4500 s^−1^M^−1^) is phosphorylated as well as NR (3900 s^−1^M^−1^), and uridine (850 s^−1^M^−1^) is within 5-fold of NR. The fact that Nrk1 and Nrk2 are distinct from uridine/cytidine kinases [19] is underscored by the poor cytidine monophosphate-forming activity of each enzyme.

### Structural Basis of Substrate Specificity

To understand the basis for substrate specificity of human Nrk1, we prepared a selenomethionyl form of human Nrk1 and grew single crystals of a complex of the enzyme with Mg^2+^·ADP. A crystal, which had the symmetry of C222_1_, was subjected to 0.9793-Å synchrotron X-radiation, and produced nearly complete diffraction data to 1.9-Å resolution (Table 2). Single-wavelength anomalous scattering [23] allowed location of Se sites [24] and phasing [25] to produce an interpretable experimental electron density map of the Nrk1 monomer prior to model building. The 1.95-Å refined protein model includes residues 1–82 and 92–189 of the 199–amino acid polypeptide, plus ADP, Mg^2+^, and 72 water molecules with B factors between 9 and 37 Å^2^.

**Table 2:**
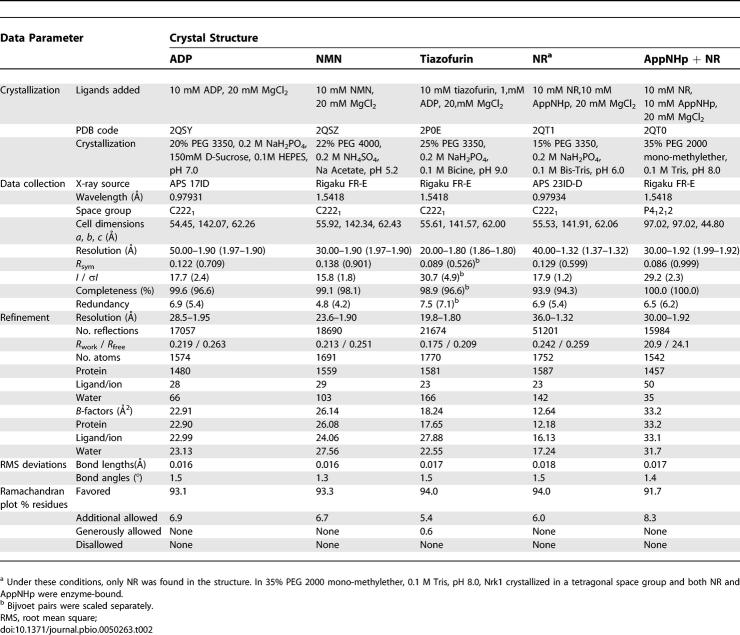
Crystallization, Data Collection, and Refinement

**Figure 1:**
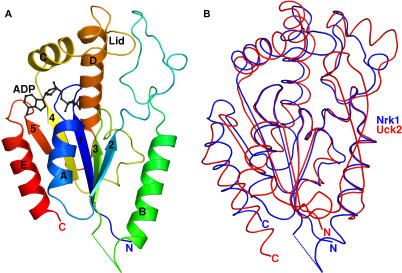
Nrk1 Is a Rossmann Fold Metabolite Kinase (A) Ribbon diagram of Nrk1 bound to ADP with rainbow coloring from the N terminus (violet) to the C terminus (red). Helices A through E, strands 1 through 5, and the lid domain are indicated. (B) Structural superposition of Nrk1 (blue) with human Uck2 (red).

As shown in Figure 1A, Nrk1 consists of a five-stranded β sheet flanked on one side by α helices, E and A, and on the other side by helix B. Additionally, the monomeric enzyme contains a lid domain consisting of helix C and D connected by a 12– amino acid loop. The five-stranded sheet is entirely parallel and is formed from strands 2, 3, 1, 4, and 5 in the primary sequence. Earlier [4], we detected sequence similarity with uridine/cytidine kinase and pantothenate kinase. Indeed, the DALI structural similarity server [26] revealed Nrk1 to be a structural homolog of a variety of Rossmann fold-containing metabolite kinases including human uridine/cytidine kinase 2 Uck2 [27], E. coli pantothenate kinase panK [20], Bacillus stearothermophilus adenylate kinase [28] and E. coli gluconate kinase [29]. A structural superposition of Nrk1 and Uck2 is provided in Figure 1B.

**Figure 2:**
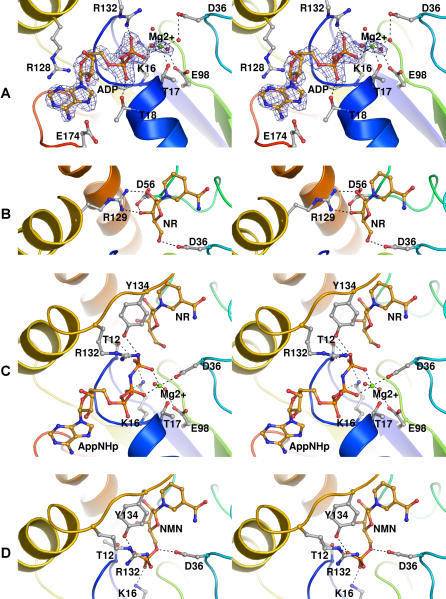
Nucleoside and Nucleotide Recognition by Nrk1 Stereo diagrams of Nrk1 are provided in a consistent orientation using the main chain backbone colors from Figure 1A. (A) ADP•Mg^2^ product complex with the corresponding difference electron density map, contoured at 3.0 Å. (B) NR substrate complex. (C) Bisubstrate analog complex of AppNHp•Mg^2^ plus NR. (D) NMN product complex.

The ADP-binding site, including P-loop [30] sequence Gly-Val-Thr-Asn-Ser-Gly-Lys-Thr (residues 10–17), is shown in close-up in Figure 2A. The guanidino group of Arg132, the ɛ amino group of Lys16, and the hydroxyl of Thr18 coordinate the β and α phosphates of ADP, whereas the hydroxyl of Thr17, a β phosphate oxygen, and four well-ordered water molecules coordinate the magnesium ion. The adenine ring of ADP lies between Arg128 and Glu174. Accounting for the ATP/GTP nonspecificity of Nrk1, the 2 carbon of adenine is solvent exposed such that the 2 amino group of guanine would not appear to preclude binding in the same manner. However, in the ATP-specific Nrk2 sequence, Glu174 is replaced with Arg. Indeed, in a large study of amino acid propensity in adenine and guanine-binding sites, Arg was found to be localized at adenine sites and to be largely excluded from guanine sites [31].

To determine the nature of nucleoside phosphorylation by Nrk1, we co-crystallized the Nrk1 enzyme with NR, NR·Mg^2+^·AppNHp, NMN, and tiazofurin, and performed molecular replacement and refinement to obtain high-resolution models. Despite the typical hinge motions identified in Rossmann fold-containing metabolite kinases upon substrate-binding [32], Nrk1 was neither opened nor closed by any ligands examined. Pairwise comparisons of the α carbon coordinates indicated that all structures are within a root mean square difference of less than 0.4 Å. As shown in Figure 2B, which is derived from a 1.32-Å crystal structure, NR is bound with the 2′ and 3′ hydroxyl groups recognized by bidentate interactions from Asp56 and Arg129 and with the carboxylate of Asp36 accepting an apparent hydrogen bond from the NR 5′ hydroxyl. Such a hydrogen bond could serve to activate the 5′ oxygen toward bond formation with the γ phosphorous atom of a bound ATP substrate.

As shown in Figure 2C, in the 1.92-Å refined crystal structure of Nrk1 bound to the hydrolysis-resistant ATP analog AppNHp with Mg^2+^ and NR, the γ phosphate—recognized by side chains of Thr12, Lys16, Tyr134, and Arg132—is positioned for potential in-line transfer to the 5' oxygen of NR. In this structure, the carboxylate of Asp36 is a direct Mg^2+^ ligand. In the NMN product complex (Figure 2D), all four side chains “formerly” associated with the γ phosphate of AppNHp are associated with the α phosphate of NMN, suggesting that these residues may be optimally aligned to stabilize a putative pentacoordinate phosphorane transition state that is resolved either by collapse to ATP + NR or by the formation of ADP + NMN products.

### Essential Carboxylates at the Nrk1 and Nrk2 Active Sites

**Figure 3:**
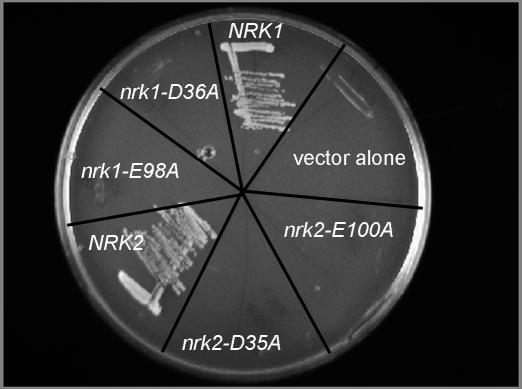
Active Site Requirement of Nrk1 and Nrk2 The conserved carboxylates of human Nrk1, Asp36 and Glu98, and the corresponding carboxylates of Nrk2 are required for biological activity in yeast.

In the NR (Figure 2B) and NMN (Figure 2D) substrate and product complexes, Asp36 is oriented toward the 5′' oxygen, suggesting a role in activating the acceptor oxygen and promoting bond formation. In the absence of NR or NMN and in the presence of the ADP product (Figure 2A), Asp36 stabilizes a Mg^2+^-associated water molecule. Curiously, Asp36 has yet a third unique conformation in the inactive bi-substrate analog complex (Figure 2C). In addition, Glu98 appears to have a key role in organizing a stable water ligand of Mg^2+^. To test the hypothesis that Asp36 and Glu98 (residues 35 and 100 in Nrk2) might be essential for function, we constructed human *nrk1-D36A, nrk1-E98A, nrk2-D35A,* and *nrk2-E100A* alleles for evaluation in yeast. These mutants, alongside wild-type *NRK1* and *NRK2* controls, were introduced into yeast strain BY278 in which *NRK* alleles were expressed from the *GAL1* promoter on a *LEU2* plasmid, the endogenous *NRK1* gene was deleted, and a *QNS1* gene was provided on a *URA3* plasmid. In this system, a functional *NRK* gene allows a yeast cell grown in the presence of 10 μM NR to lose the *QNS1* gene with associated *URA3* marker, as scored by resistance to 5-fluoro-orotic acid [4]. As shown in Figure 3, the conserved Asp and Glu residues are required for function of Nrk enzymes in vivo. To exclude the possibility that the conserved Glu residues are required for folding and are potentially dispensable after protein biosynthesis, we expressed and purified nrk1-E98A and nrk2-E100A mutant proteins in E. coli. As shown in Figure S1, the conserved Glu is not required for soluble expression, accumulation, purification, or concentration. Although nrk1-E98A and nrk2-E100A proteins behaved precisely as did wild-type enzymes in purification, their activity in ATP-dependent phosphorylation of NR was below the level of detection of our assay. Thus, Asp36 (Asp35 in Nrk2) and Glu98 (Glu100 in Nrk2) are essential residues for function in vivo. The demonstrated post-biosynthetic role for the conserved Glu and the conserved active-site positions of Glu and Asp strongly argue for roles in catalysis.

### Base Recognition in the Nrk1 Nucleoside-Binding Site Excludes Uridine but Supports NaR Phosphorylation

**Figure 4:**
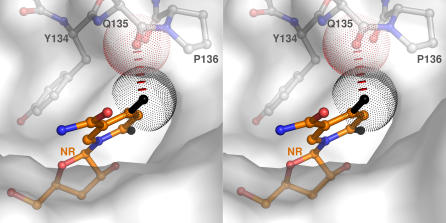
NR/Uridine Discrimination by Nrk1 In stereo, the 2- and 4-oxy functions (black) of a hypothetical uridine substrate are shown superimposed onto NR coordinates in the Nrk1 NR co-crystal structure. At a distance of 2.55 Å, the 4 oxygen would be in van der Waals conflict with the carbonyl oxygen of Gln135, which is in a *cis* peptide linkage with Pro136.

Data in Table 1 show that Nrk1 has strong specificity for nucleosides containing a carboxamide group two bond lengths away from N1, such as NR and tiazofurin, and features that may discriminate against the 2- and/or 4-substitutions found in cytidine and uridine. Indeed, in crystal structures of Nrk1 bound to NR and NMN, it is clear that the 4-amino group of cytidine or the 4-oxy group of uridine could not be accommodated without rearrangement, because these constituents would clash with the carbonyl oxygen of Gln135, which is in a *cis* peptide linkage with Pro136. This unique backbone conformation is unlikely to be conserved by the uridine-accepting Nrk2 enzyme, which has a Thr-Val sequence in this position, likely to be in the typical *trans* conformation. The van der Waals clash between a modeled 4-oxy group of uridine and the Gln135 carbonyl oxygen is shown in Figure 4.

**Figure 5:**
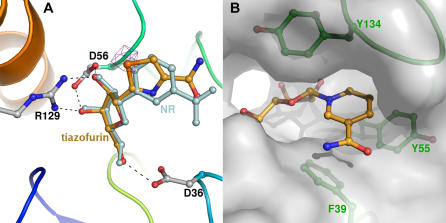
NR and Tiazofurin Recognition by Nrk1 (A) Coordinates of tiazofurin, in yellow, are superimposed with those of NR in isomorphous crystal structures. The sulfur atom of tiazofurin was localized by the 3 Å anomalous difference Fourier map, contoured in purple at 2.7 Å. (B) A surface representation of the Nam binding-site of NR reveals no specific recognition of the carboxamide group, which is exposed to solvent.

The carboxamide-containing preferred nucleoside substrates of Nrk1 are tiazofurin and NR. As shown in Figure 5A, the two nucleosides are bound almost superimposably by Nrk1. The pyridine and thiazole moieties of NR and tiazofurin, respectively, are stacked between the phenol rings of Tyr55 and Tyr134. An additional aromatic interaction with Phe39 allows the polar carbonyl oxygen and amino groups of NR and tiazofurin to be exposed to solvent (Figure 5B). Because carbonyl oxygen and amino groups are isosteric at 1.9-Å resolution, we looked for an electrostatic interaction that might uniquely orient the carboxamide function of NR and tiazofurin, and found no interacting residue within hydrogen-bonding distance. We therefore considered the possibility that Nrk enzymes might phosphorylate the isosteric but nonisoelectronic NR analog, NaR. Consequently, NaR was synthesized and examined as an in vitro substrate of Nrk1 and Nrk2. As shown in Table 1, NaR is phosphorylated by Nrk1 and Nrk2 with highly similar kinetics with respect to those for NR phosphorylation. In assays of each human enzyme, NaR is favored over NR by slight *K*_M_ advantages offset by slight *k*_cat_ disadvantages. In *k*_cat_/*K*_M_ terms, Nrk1 has 60% of the activity and Nrk2 has 138% of the activity with NaR versus NR. Thus, human Nrk1 and human Nrk2 are dual-specificity, NR and NaR kinases.

### NaR Is a Novel NAD^+^ Precursor Utilized via the Nrk and Preiss-Hander Pathways

**Figure 6:**
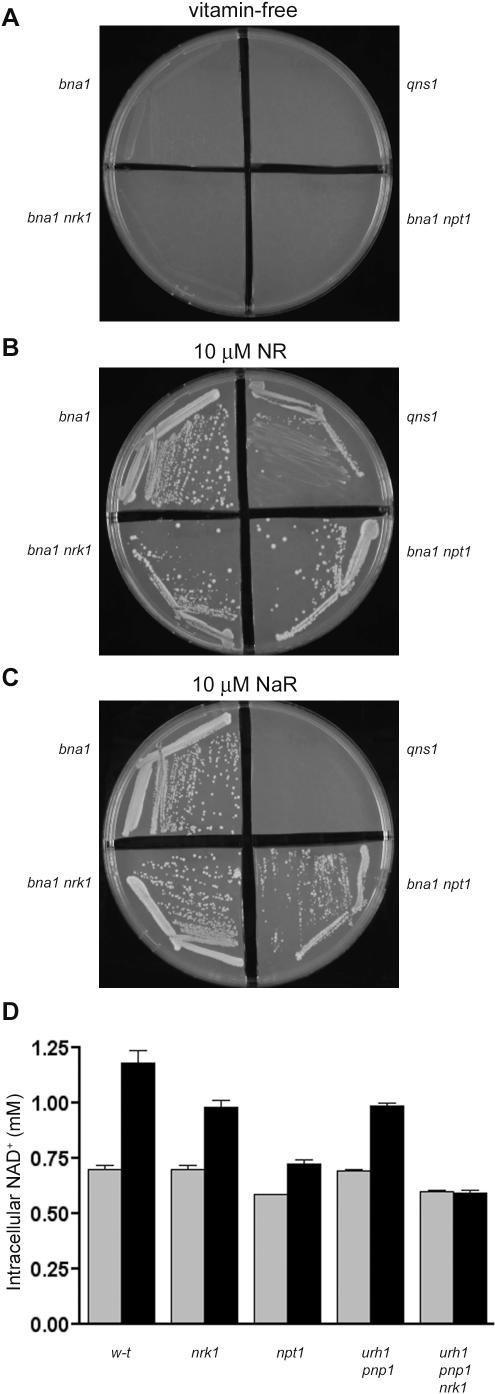
NaR Utilization In Vivo In (A), the vitamin requirement of de novo mutant *bna1* and glutamine-dependent NAD^+^ synthetase mutant *qns1* is illustrated. The fact that *bna1*, *bna1 nrk1*, *bna1 npt1*, *and qns1* strains are satisfied by addition of NR is shown in (B). In (C), the vitamin activity of NaR is demonstrated for the de novo mutant *bna1*, even when either the Nrk pathway or the Preiss-Handler pathway is mutationally inactivated by *nrk1* or *npt1* mutation, respectively. Establishing the uniqueness of NaR as a vitamin, NaR fails to support the growth of *qns1*. In (D), the intracellular NAD^+^ concentration is calculated for wild-type, *npt1*, *nrk1*, *urh1 pnp1*, and *nrk1 urh1 pnp1* mutants in vitamin-free (gray bars) and in vitamin-free media supplemented with 10 μM NaR (black bars). The unique lack of utilization by the *nrk1 urh1 pnp1* strain shows that NaR makes use of Nrk1, Urh1, and Pnp1 for conversion to NAD^+^.

**Figure 7:**
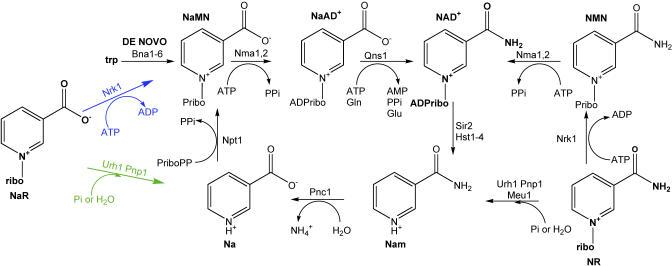
NAD^+^ Metabolism in Yeast: Two New Pathways to NaMN Previously reported NAD^+^ metabolic pathways are shown in black[15]. In blue and green, respectively, are Nrk1-dependent and Nrk1-independent routes from NaR to NaMN.

The steric complementarity of Nrk1 with NR and the lack of electrostatic exclusion of NaR by human Nrk1 and Nrk2 suggested that NaR might be a synthetic NAD^+^ precursor vitamin. Should NaR be utilized by yeast cells, it would be conceivable that NaR is a previously unrecognized metabolite, such that the utility of NaR salvage might have played a role in the evolution of Nrk specificity. Additionally, our discovery of Nrk1-dependent [4] and Nrk1-independent [15] NR utilization suggested that, should NaR support the vitamin requirement of de novo pathway–deficient yeast cells, there could be two different metabolic pathways for NaR utilization. As shown in Figure 6A and 6B, the *bna1* mutant in de novo biosynthetic enzyme 3-hydroxyanthranilic acid dioxygenase is a Na auxotroph [33] that can also be supported by 10 μM NR, thus providing an assay for vitamin activity of NaR. As reported earlier [4], NR can bypass the requirement of glutamine-dependent NAD^+^ synthetase, Qns1 [21]. Also shown in Figure 6B, NR keeps a *bna1* mutant alive in two different ways because cells have two NR salvage pathways [15]. The first NR salvage pathway goes through Nrk1, which allows NR-dependent viability in a *bna1* mutant deleted for Npt1, which is the Na phosphoribosyltransferase. The second NR salvage pathway depends on the NR-splitting activities of Urh1 and Pnp1, followed by nicotinamidase and Npt1 activities. This pathway allows a *bna1 nrk1* double mutant to retain viability. The two NR salvage pathways are schematized in the lower right section of Figure 7.

As shown in Figure 6C, 10 μM NaR can also be used by *bna1* mutants, establishing that NaR is a transportable NAD^+^ precursor vitamin. Genetic control over NAD^+^ biosynthesis in the yeast system allowed us to establish that NaR is not simply used as Na, not contaminated by or converted to NR, and is used via a unique set of enzymes including Nrk1, Urh1 and Pnp1, and Qns1. If NaR were merely a source of Na, the *bna1 npt1* mutant would fail to grow on NaR. However, Figure 6C clearly shows that NaR supports the growth of *bna1 npt1* mutant yeast cells. Moreover, if NaR were either contaminated by NR or converted to NR by any cellular process, then NaR would support the growth of the *qns1* mutant. As shown in Figure 6C, NaR fails to support the growth of the *qns1* mutant. Whereas NaR shares with NR the ability to be utilized by *bna1 npt1* and *bna1 nrk1* mutants, the Qns1 requirement for NaR indicates that NaR metabolites must flow through nicotinic acid mononucleotide (NaMN) and nicotinic acid adenine dinucleotide (NaAD) as schematized in Figure 7.

Vitamin activity of NaR in *bna1 npt1* and *bna1 nrk1* mutant strains can be explained by two pathways for NaR utilization. NaR is phosphorylated by Nrk1 with highly similar kinetics to those of NR (Table 1). Thus, phosphorylation of NaR by Nrk1 produces NaMN in a pathway that is independent of Npt1. By analogy with the recently described Nrk1-independent NR utilization pathway, we hypothesized that NaR is a substrate of the nucleoside hydrolase and nucleoside phosphorylase activities of Urh1 and Pnp1, which are responsible for virtually all Nrk1-independent NR salvage [15]. However, whereas yeast Nrk1-independent NR salvage requires nicotinamidase, the corresponding pathway for NaR would simply produce Na from NaR, which would be salvaged by the Preiss-Handler pathway [34], consisting of Na phosphoribosyltransferase Npt1 [7], Nma1,2, and Qns1.

To test the hypothesis that NaR utilization depends on Nrk1, Urh1, and Pnp1, i.e., the same enzymes that initiate NR salvage [15], we grew wild-type, *npt1* mutant, *nrk1* mutant, *urh1 pnp1* mutant, and *nrk1 urh1 pnp1* mutant cells in vitamin-free media and in vitamin-free media supplemented with 10 μM NaR. As shown in Figure 6D, NaR elevates NAD^+^ levels in all strains except the *nrk1 urh1 pnp1* mutant. In the wild-type strain, NaR elevated intracellular NAD^+^ from 0.70 ± 0.04 mM to 1.18 ± 0.10 mM, an increase of 480 μM. This can be compared with the 1.21 mM increase in intracellular NAD^+^ that is produced by growing wild-type cells in 10 μM NR [15]. Elimination of Nrk1 or both Urh1 and Pnp1 produced an identical 40% decline in the ability of NaR to elevate NAD^+^. In the *nrk1* mutant, NAD^+^ was elevated from 0.70 ± 0.03 mM to 0.99 ± 0.05 mM, whereas NAD^+^ was elevated from 0.70 ± 0.01 mM to 0.99 ± 0.02 mM in the *urh1 pnp1* double mutant. Two strains, namely the *npt1* mutant (0.59 ± 0.001 mM), which is deficient in the NAD^+^ salvage necessitated by Sirtuin activity [7], and the *nrk1 urh1 pnp1* mutant (0.60 ± 0.02 mM), which is deficient in NR utilization and salvage [15], have baseline NAD^+^ concentrations in vitamin-free media that are 110 μM and 100 μM lower than those of the other strains, respectively. However, whereas the *npt1* mutant was increased to 0.73 ± 0.03 mM with addition of NaR, the triple mutant in NR utilization was unable to obtain an increase in NAD^+^ concentration in response to NaR (0.59 ± 0.02 mM).

Thus, NaR is a synthetic vitamin precursor of NAD^+^ that is phosphorylated by Nrk enzymes in vitro (Table 1) and utilized in vivo (Figure 6). In vivo utilization of NaR is not limited to the Nrk pathway producing NaMN, because NaR can fulfill the vitamin requirement of a *bna1 nrk1* mutant, and NaR can elevate NAD^+^ in cells without Nrk1. Just as NR salvage goes through Nrk and Urh1/Pnp1 pathways [15], the nucleoside-splitting activities of Urh1 and Pnp1 and Nrk1 must be eliminated to block NaR utilization. Yeast NAD^+^ biosynthetic pathways updated to include NaR utilization are schematized in Figure 7.

## Discussion

The experiments performed herein establish that Asp36 and Glu98 have essential roles in Nrk function. Structures of Nrk1 bound to adenosine nucleotides and pyridine and thiazol nucleoside substrates provided information of the basis for ATP/GTP nondiscrimination and pyrimidine exclusion by Nrk1. Structural analysis of Nrk2 is expected to shed light on how Nrk2 excludes GTP and phosphorylates uridine.

It has been established that no yeast enzyme can substitute for Nrk1 in conversion of NR to NMN in vivo [4]. Moreover, the postulated role for Nrk enzymes in phosphorylating NR-mimetic prodrugs has created expectations for strong specificity in carboxamide recognition. Structures of Nrk1 with NR and tiazofurin, however, indicated that the nucleosides are recognized by polar interactions with the 2′, 3′, and 5′ hydroxyl groups and aromatic interactions with the base. Whereas there are steric clashes that would appear to destabilize the 4-substitutions found in cytosine and uracil (Figure 4), there is steric complementarity for the 3 carboxamide moiety in NR (Figure 5B). Recognizing the absence of an enzyme feature that would specifically orient the carboxamide group, we synthesized NaR and discovered that both Nrk1 and Nrk2 are dual-specificity NR and NaR kinases (Table 1). To determine whether this specificity is merely a biochemical curiosity or might indicate another cellular function, we determined whether yeast cells deficient in de novo NAD^+^ biosynthesis can use NaR in vivo. According to genetic data presented in Figure 6 and schematized in Figure 7, NaR is used via Na salvage and via Nrk1-dependent production of NaMN. The Nrk-dependent and Nrk-independent salvage of NaR is precedented by the processes by which NR is utilized in yeast cells. NR is utilized in an Nrk-dependent process that does not depend on the glutamine-dependent NAD^+^ synthetase [4]. However, NR is also utilized in a process that depends on NR splitting by Urh1, Pnp1 and Meu1, the Pnc1 nicotinamidase, and the Preiss-Handler pathway [15]. The unique feature of NaR as an in vivo substrate of the Nrk pathway is that NaR requires Nrk and NAD^+^ synthetase.

The importance of the dual specificity of Nrk enzymes at phosphorylating NR and NaR is three-fold. First, there are active programs to design prodrugs of NAD^+^-antagonistic compounds such as TAD and benzamidine adenine dinucleotide. It has been assumed that such prodrugs must not stray far from NR to allow phosphorylation, adenylylation, and inhibition of the target dehydrogenases [35]. However, the discovery that Nrk1 and, apparently, Nrk2 exhibit steric but not electrostatic recognition of the carboxamide group will allow a wider range of prodrugs to be synthesized and evaluated.

Second, the abilities of yeast to use NaR and of human Nrk enzymes to phosphorylate NaR in vitro suggest that NaR might be useful as a vitamin precursor to NAD^+^. However, because Nrk-independent salvage requires expression of all three Preiss-Handler enzymes, Nrk-independent utilization of NaR would amount to supplementing with a very expensive form of Na. This is impractical because Na is already readily available in the diet and may be tissue-limited by the expression of Na phosphoribosyltransferase. Because maturation of NaR to NAD^+^ through the Nrk pathway requires the activity of NAD^+^ synthetase, NaR might be more tissue-restricted than NR or constitute a slower-release niacin-equivalent than NR. Thus, it is conceivable that NaR could be a useful supplement, particularly if it largely evades Nrk-independent phosphorolysis to Na or, as suggested for NR [15], if NaR phosphorolysis can be inhibited, presumably with a Pnp inhibitor.

Finally, the biotransformation of NaR by the two NR salvage systems in yeast prompts us to ask whether NaR might be an endogenous metabolite, such that the utility of NaR phosphorylation could have played a role in maintaining dual NR/NaR substrate specificity. NR was initially characterized as a compound produced in the laboratory and found in milk that can provide for *qns1*-independent yeast cell growth when added exogenously [4]. Exogenously applied NR protects against transection-induced degeneration of murine dorsal root ganglion neurons [16]. In the yeast system, exogenously applied NR increases NAD^+^ levels, Sir2 function, and replicative life span [15]. Additionally, the yeast study provided evidence for an endogenous NAD^+^ catabolic process that creates a requirement for NR salvage enzymes to maintain NAD^+^ levels [15]. By deleting the *NRK1*, *URH1*, and *PNP1* genes, which account for virtually all NR utilization through both the Nrk-dependent and the Nrk-independent pathways, we showed that there is a significant (0.8 mM) deficiency in NAD^+^ levels in cells grown in standard media, which does not contain any NR [15]. These data strongly argue for an endogenous process that produces NR and/or NaR at the expense of NAD^+^. Indeed, because *npt1* and *nrk1 urh1 pnp1* mutants have the same deficiency in baseline NAD^+^ levels in vitamin-free media (Figure 6D), we suggest that the rate of NAD^+^ catabolism to NR and/or NaR is comparable to the rate of Sirtuin-dependent consumption of NAD^+^ to Nam.

## Materials and Methods

### Enzyme purification and characterization.

His-tagged human Nrk1 and Nrk2 proteins were expressed and purified from E. coli strain BL21(DE3) as described [4]. Kinetic analyses were performed in 20 mM HEPES, pH 7.5, 100 mM NaCl, 5 mM MgCl_2_ with 1 mM ATP or GTP as phosphodonor and with varying concentrations of nucleoside substrates. Reactions were initiated by Nrk1 or Nrk2 enzyme sufficient to convert 1% to 10% of the input nucleoside to nucleoside monophosphate in 30 min incubations at 37 °C. Products were quantified by anion exchange high-performance liquid chromatography (HPLC) as described [4] and kinetic parameters were determined from Lineweaver-Burke plots.

### Crystallization, structure determination, and refinement.

Nrk1 (30 mg/ml) was crystallized by 1:1 sitting drop vapor diffusion (18 °C) against the reservoir solutions listed in Table 2. Crystals were cryo-protected in 1:1 paratone and mineral oil. Diffraction data (Table 2) were reduced to intensities with the HKL2000 suite [36], and the first Nrk1 structure was solved de novo as described in the text. ARP/wARP [37] was used for model building, and PHASER [38] was used for molecular replacement of subsequent Nrk1 structures. Geometric restrains for NR, NMN, and tiazofurin were generated on the PRODRG server [39]. Restrained refinement using REFMAC [40], geometric validation using MOLPROBITY [41], and manual rebuilding using COOT [42] were performed iteratively until convergence (Table 2). Coordinate alignments were performed by secondary structure matching [43] within COOT. Molecular graphics were produced with PyMOL [39]. Structure factors and coordinates have been deposited in the Protein Data Bank.

### Synthesis of NaR.

Trimethylsilyl trifluoromethanesulfonate (1.039 g, 4.4 mmol; Sigma-Aldrich; http://www.sigmaaldrich.com) was slowly added to ethyl nicotinate (0.9 ml, 6.6 mmol; Sigma-Aldrich) and 1,2,3,5-tetra-O-acetyl-β-D-ribofuranose (1.4 g, 4.4 mmol; Sigma-Aldrich) in 50 ml anhydrous methylene chloride at room temperature, stirred under argon. The mixture was heated to reflux for 8 h. TLC (CH_2_Cl_2_: MeOH: TEA=5: 0.3: 0.05) stained with 10% H_2_SO_4_ in MeOH showed the disappearance of the ribofuranose and appearance of the presumed product, 2′, 3′, 5′-triacetyl ethyl NaR in a single spot at lower mobility relative to the front. After evaporation of methylene chloride, product (25 mg, 0.05 mmol) was added into 0.9 ml of 312 mM NaOEt in EtOH on ice to form O-ethyl β-NaR. After mixing well, the reaction was stored at −20 °C overnight. The reaction was quenched with addition of acetic acid to neutralize the pH. After organic solvent was removed in vacuum, the residue was dissolved in water and extracted with cyclohexane to remove organic impurities. The aqueous phase was then concentrated 10-fold, made to 150 mM in phosphate buffer, and provided with 10 μl of pig liver esterase (13 units; Sigma-Aldrich) to release NaR in a 25 °C overnight incubation. NaR was purified by C-18 HPLC. NaR was assayed by MALDI MS, in positive ion detection mode, and was observed as the protonated molecular ion (predicted mass-to-charge ratio *m*/*z*= 256.08, observed *m*/*z*= 256.1). Other assignable fragmented ions detected included protonated Na. The entire *m*/*z* spectrum (% peak height) was 256.1 (1.81%), 228.0 (48.5%), 207.1 (25.4%), 146.1 (10.3%), and 124.0 (13.9%). We used a molar extinction coefficient of 6411 cm^−1^ (260 nm) for NaR and 4305 cm^−1^ (259 nm) for NR.

### S. cerevisiae strains, plasmids, and media.

Yeast strain BY278, which contains *qns1* deletion covered by plasmid pB175 *(QNS1* and *URA3)* and which contains *nrk1* deletion, has been described [4]. pB450 and pB459, which are *LEU2* plasmids for expression of human *NRK1* and *NRK2* cDNAs under *GAL1* promoter control [4], were used as templates for site-directed mutagenesis to produce *nrk1-D36A* (pHC12), *nrk1-E98A* (pHC10), *nrk2-D35A* (pHC13), and *nrk2-E100A* (pHC11). BY278 was transformed with each plasmid and the empty p425*GAL1* control. After passage on galactose media, transformants were streaked on synthetic complete, galactose media with 5-fluoroorotic acid and 10 μM NR [4] to score the function of *NRK* alleles. Isogenic strains for the NaR utilization study were BY165-1d *(qns1)* [4], KB046 (*bna1* in the deletion consortium background [44]), KB056 (*nrk1* deleted from KB046), and JS949 (*bna1 npt1*, a gift of Jeffrey S. Smith, University of Virginia, United States). The four strains were grown in synthetic media with 3 μM Na plus 10 μM NR, washed in saline, and then cultured to exhaustion in vitamin-free media [15]. To assay utilization of NR or NaR, strains grown to exhaustion in vitamin-free media were streaked on vitamin-free synthetic media supplemented with 10 μM NR or NaR and photographed after 3 d at 28 °C. NAD^+^ measurements were performed as described [15] with isogenic yeast strains grown in vitamin-free media and vitamin-free media supplemented with 10 μM NaR to an optical density (OD)_600 nm_ of 1. Strains were wild-type BY4742, KB009 (*nrk1* in the deletion consortium background [44]), KB008 (*npt1* in the deletion consortium background [44]), PAB047 (*urh1 pnp1* [15]) and PAB038 (*urh1 pnp1 nrk1* [15]).

## Supporting Information

Figure S1 Expression and Activity Analysis of Nrk Active-Site Glutamate Mutants His-tagged human wild-type Nrk1 and Nrk2 and glutamate mutants were expressed and purified from E. coli by immobilized cobalt affinity chromatography. The expression and purification of wild-type Nrk2 and Nrk2-E100A were analyzed by SDS-PAGE. Both Nrk2 and Nrk2-E100A exhibited high-level overexpression in the total cell lysate and virtually identical behavior upon chromatography. Relative specific activities were calculated for Nrk1, Nrk1-E98A, Nrk2, and Nrk2-E100A. Reactions contained 1 mM ATP as phosphodonor and 1 mM NR as nucleoside acceptor. Reactions were incubated for 30 min at 37 °C. NMN was quantified by anion exchange HPLC.
(74 KB DOC)

### Accession Numbers

The Swiss-Prot (http://www.ebi.ac.uk/swissprot) accession numbers for proteins in this paper are: human MTAP (Q13126); human Nrk1 (Q9NWW6); human Nrk2 (Q9NPI5); human NP (P00491); human Uck2 (Q9BZX2); S. cerevisiae Bna1 (P47096); S. cerevisiae Meu1 (Q07938); S. cerevisiae Npt1 (P39683); S. cerevisiae Nrk1 (P53915); S. cerevisiae Pnc1 (P53184); S. cerevisiae Pnp1 (Q05788); S. cerevisiae Qns1 (P38795); S. cerevisiae Urh1 (Q04179); E. coli gntK (P46859); E. coli panK (P0A615); and B. stearothermophilus adk (P27142). The Protein Data Bank (PDB) (http://www.rcsb.org/pdb) accession numbers for human Nrk1 are 2QSY, 2QT1, 2QT0, 2QSZ, and 2P0E; for human Uck2 is 1UJ2.

## Abbreviations

AppNHp: adenosine-5′-[(β,γ)-imido]triphosphate
Na: nicotinic acid
NaAD: nicotinic acid adenine dinucleotide
Nam: nicotinamide
NaMN: nicotinic acid mononucleotide
NaR: nicotinic acid riboside
NMN: nicotinamide mononucleotide
NR: nicotinamide riboside
Nrk: nicotinamide riboside kinase
