# Supplementary material for: Nicotinamide Riboside Kinase Structures Reveal New Pathways to NAD+
Source: PLoS Biol. 2007 Oct 2;5(10):e263. doi: 10.1371/journal.pbio.0050263 (PMC1994991; doi:10.1371/journal.pbio.0050263)
Supplement: Supplementary file 1 [file pbio.0050263.sg001.doc]

Relative Specific Activity of Nrk1-E98A and Nrk2-E100A

|  | Nrk1 | Nrk1-E98A | Nrk2 | Nrk2-E100A |
| --- | --- | --- | --- | --- |
| Specific Activity | 100% | < 0.8% | 100% | < 0.9% |
